# The global and regional prevalence of restless legs syndrome among adults: A systematic review and modelling analysis

**DOI:** 10.7189/jogh.14.04113

**Published:** 2024-06-07

**Authors:** Peige Song, Jing Wu, Jin Cao, Weidi Sun, Xiaoyu Li, Tianjing Zhou, Yaojia Shen, Xiao Tan, Xinxin Ye, Changzheng Yuan, Yajie Zhu, Igor Rudan, Global Health Epidemiology Research Group (GHERG)

**Affiliations:** 1 Department of Big Data in Health Science, School of Public Health and The Second Affiliated Hospital, Zhejiang University School of Medicine, Hangzhou, China; 2 The Key Laboratory of Intelligent Preventive Medicine of Zhejiang Province, Hangzhou, Zhejiang 310058, China; 3 School of Public Health, Zhejiang University School of Medicine, Hangzhou, China; 4 Department of Sociology, Tsinghua University, Beijing, China; 5 Department of Epidemiology and Biostatistics, School of Public Health, Peking University Health Science Center, Beijing, China; 6 Department of Maternal and Child Health, School of Public Health, Peking University, Beijing, China; 7 Department of Sports and Exercise Science, College of Education, Zhejiang University, Hangzhou, Zhejiang, China; 8 School of Information Science and Technology, Hangzhou Normal University, Hangzhou, China; 9 Centre for Global Health Research, Usher Institute of Population Health Sciences and Informatics, University of Edinburgh, Edinburgh, Scotland, UK

## Abstract

**Background:**

Restless legs syndrome (RLS) is a prevalent neuro-sensory disorder that impairs quality of life. In this systematic review and modelling study, we estimated the global and regional prevalence of RLS and its associated factors.

**Methods:**

We searched PubMed, Embase, and Medline for population-based studies on RLS prevalence published up to 12 November 2023. The included studies reported prevalence using the International Restless Leg Syndrome Study Group's (IRLSSG) minimal diagnostic criteria without limitations on frequency, duration, or severity. We applied a multilevel multivariable mixed-effects meta-regression to generate the age-specific and sex-specific prevalence of RLS for high socio-demographic index (H-SDI) and low and middle socio-demographic index (LM-SDI) regions. We pooled odds ratios (ORs) for RLS associated factors using random-effects models. Finally, we derived the regional prevalence and cases of RLS based on an associated factor-based model.

**Results:**

From 52 articles across 23 countries, the global RLS prevalence in 2019 was estimated to be 7.12% (95% confidence interval (CI) = 5.15–9.76) among adults 20–79 years of age, equating to 356.07 million (95% CI = 257.61–488.09) affected individuals. Prevalence was similar in H-SDI (7.29%; 95% CI = 5.04–10.41) and LM-SDI (7.10%; 95% CI = 5.16–9.70) regions, with the majority of cases in LM-SDI countries (323.06 million; 90.73%). Europe had the highest (7.60%; 95% CI = 5.44–10.52) and Africa the lowest regional prevalence (6.48%; 95% CI = 4.70–8.87). The Western Pacific Region, meanwhile, had the most cases (111.91 million; 95% CI = 80.93–153.42). Factors positively associated with RLS included advanced age (OR = 1.13; 95% CI = 1.04–1.24), smoking (OR = 1.46; 95% CI = 1.29–1.64), depression (OR = 1.71; 95% CI = 1.26–2.32), and diabetes (OR = 1.54; 95% CI = 1.19–1.97).

**Conclusions:**

A considerable global burden of RLS exists. Effective strategies are needed to increase awareness and optimise resource allocation to address this often-overlooked condition. High-quality epidemiological investigations employing standardised and rigorous criteria for RLS are essential for addressing RLS burden more effectively.

**Registration:**

PROSPERO: CRD42020161860.

Restless legs syndrome (RLS), or Willis-Ekbom Disease (WED), is increasingly acknowledged as a significant neuro-sensory disorder with substantial public health implications [1]. It manifests as an uncontrollable urge to move one’s legs, frequently accompanied by discomfort and paraesthesia, worsening during rest and improving with movement [1]. These symptoms, peaking in severity during evening hours, can range from sporadic, mild discomfort to persistent and severe disruption, significantly impairing sleep, quality of life, and mental health, particularly when diagnosis and treatment are inadequate [1–4]. In extreme cases, RLS is associated with increased risks of depression, suicidal tendencies, and self-harm, underscoring its potential impact on a broader range of health outcomes [5,6]. RLS can present alone or with comorbid conditions such as cardiovascular disease, diabetes, and arterial hypertension, complicating diagnosis and management [7,8].

For many years, RLS was underestimated as a rare condition, with its peculiar symptomatology and absence during wakefulness leading to misdiagnosis as a psychosomatic condition. The lack of definitive physical signs, as evidenced in nerve-conduction studies and electromyography, further contributed to this misconception. In 1995, the International Restless Leg Syndrome Study Group (IRLSSG) proposed the so-called RLS minimal diagnostic criteria, which included an urge to move one’s limbs, motor restlessness, worsening of symptoms at rest, and worsening of symptoms at night [9]. Subsequently, the 2003 National Institutes of Health/IRLSSG criteria and the revised criteria in 2014 contributed to significant milestones in RLS research [10]. These criteria set the cornerstone for evaluating the prevalence of RLS in population studies and care needs in clinical settings. Benefiting from those criteria, an increasing number of epidemiological investigations have explored the distribution of RLS in various populations, finding it to be a prevalent, treatable, yet often underdiagnosed condition, with prevalence rates ranging between 4% and 14% [4,11,12].

To set priorities for public health policy, fund public health initiatives, and develop health care planning, the prevalence of RLS in the general population needs to be determined, with highlights on variations across different socio-demographic features [3,13]. While recent efforts by Broströme et al. [14] have estimated a synthesised global RLS prevalence of 3% among the general population worldwide, significant uncertainties persist due to methodological heterogeneity in study designs (cross-sectional, cohort, and case-control), diagnostic criteria (IRLSS 2003, IRLSS 1995, and others), and participant age groups (adults and elderly) [4,14]. Furthermore, previous global estimates have not sufficiently addressed age-related variations in RLS prevalence, which hampers the design of targeted public health strategies and clinical guidelines. Furthermore, the identification of RLS risk factors remains limited by the scarcity of longitudinal studies on this topic [12,15,16], leaving a significant gap in our understanding of RLS’s aetiology from a population perspective and hampering the development of potential preventative measures [14].

In light of these considerations, we conducted a comprehensive systematic review and modelling analysis of epidemiological studies that have investigated the prevalence of RLS in the general population. By adhering to the IRLSSG minimal diagnostic criteria for defining RLS, we aimed to provide a unified, detailed, up-to-date estimation of the global and regional prevalence of RLS, with an emphasis on the variations by age, sex, and geographic region. Additionally, we sought to identify potential associated factors of RLS.

## METHODS

### Search strategy

We conducted and reported this systematic review and modelling analysis in accordance with the PRISMA guideline [17] and the Guidelines for Accurate and Transparent Health Estimates Reporting (GATHER) [18].

We comprehensively searched PubMed, Embase, and Medline for observational population-based studies published from the inception of each database up to 12 November 2023, without language or geographical restrictions. We used main terms related to RLS (i.e. ‘restless legs syndrome,’ ‘restless leg syndrome,’ ‘Ekbom syndrome,’ ‘Willis-Ekbom disease’ and ‘RLS’), alongside prevalence-related terms (i.e. ‘epidemiology’ and ‘prevalence’) and adapted the search strategy to each (Table S1 in the **Online Supplementary Document**). Additionally, we examined the reference lists of relevant reviews and included studies to supplement the bibliographic database searches.

### Study selection

We included population-based studies that reported on the prevalence rates of RLS in the general population. To be eligible, a study had to have employed either a cross-sectional or a cohort design, provided that the prevalence of RLS was assessed cross-sectionally. Moreover, these studies were required to provide prevalence rates of RLS with associated measures of uncertainty (confidence intervals (CIs) or standard errors (SEs)) or adequate data to enable the calculation of these measures (sample size and number of RLS cases). Investigations could have been organised in communities, schools, or health care facilities. Of note, investigations in health care facilities must have been conducte in the form of regular health check-ups. The investigated sample must not have been purposely selected or clearly unrepresentative of the general population, such as individuals with specific diseases or conditions like obesity. Importantly, studies needed to employ the minimal diagnostic criteria for defining RLS as outlined by IRLSSG in either their 1995 or 2003 guidelines, but should have not employed limitation of frequency, duration and/or severity. We excluded case reports, viewpoints, or opinion-based articles lacking explicit primary data.

After removing duplicates from different bibliographic databases, two authors (YS and TZ) independently screened the titles and abstracts of all identified records from the literature search. Then the same two authors assessed the eligibility of potentially relevant articles in full text against the selection criteria. Publications that were not in English or Chinese were translated using Google Translate to ensure no study was excluded due to language barriers. When multiple publications were based on the same data set, we compared them and selected the one that provided the most comprehensive results or the most recent data. Any discrepancies during study inclusion, data extraction, or quality assessment were resolved through consensus discussions involving a senior investigator (XY).

### Data extraction

Two authors (YS and TZ) independently extracted the following data from the included articles: 

− Study characteristics – author(s), year of publication, study location (country, setting (urban vs rural), and region), study design, year of investigation, sampling method, RLS case definition; 

− Population characteristics – proportion of female participants, age (mean, median, or range) of participants, sample size, number of RLS cases, and prevalence.

We also extracted the year-specific socio-demographic index (SDI), a summary measure of overall development constructed from income per capita, average years of schooling, and total fertility rate, for each country [19]. Moreover, we designated the geographic regions of study location as the African Region (AFR), the Region of the Americas (AMR), the Southeast Asia Region (SEAR), the European Region (EUR), the Eastern Mediterranean Region (EMR), and the Western Pacific Region (WPR) according to the World Health Organization (WHO) criteria. We also classified regions by development as high SDI (H-SDI) countries and low and middle SDI (LM-SDI) countries using an SDI cut-off of 0.455 [19]. For studies in which the year of investigation was not provided, we imputed it by subtracting four years from the year of publication based on the mean time lag between the year of investigation and publication where data were provided (Table S2 in the **Online Supplementary Document**). When available, we abstracted stratified prevalence data by age group, sex, setting, and geographical location. In case of censored age range, we imputed the upper or lower limit of age range using the same width as reported in other age groups in the same article. Finally, we extracted the associated factors of RLS and their corresponding fully-adjusted odds ratios (ORs) using multivariable logistic regressions.

### Quality assessment

We assessed the quality of included articles using the Quality Assessment Tool with five modules: sample population, sample size, participation rate, outcome assessment, and analytical methods. The total score, ranging from zero to ten, represented the overall bias risk of each article (Table S3 in the **Online Supplementary Document**).

### Statistical analysis

The focus of this study was on RLS defined with the minimal diagnostic criteria of IRLSSG, without limitation of a specific frequency, duration, and/or severity. To derive a robust estimation, we restricted prevalence and number of cases to the age range 20–79 years, a range for which a comprehensive number of datapoints were available.

#### Estimation of the global prevalence and cases of RLS in 2019

To best describe and fit the hierarchical structure of extracted data (i.e. multiple data points from the same study), we modelled a multilevel mixed-effects meta-regression to establish the relation between age, sex, and RLS prevalence. To enable the inclusion of zero cases as reported, we replaced zero cells with a value of 0.0005. We controlled the clustering effect of data points from the same study or country by adding the study and country identification into the regression model as the random effect (*u_i_*). We first examindd the associations of cluster-level variables using an age- and sex-adjusted meta-regression (Table S4 in the **Online Supplementary Document**), where we found the prevalence of RLS to be positively associated with age, and lower among males (than among females) and in WPR (than in EUR). In the multivariable models, we included age and sex in the regression models of RLS prevalence. The nonlinear relation between age and RLS prevalence was modelled using a restricted cubic spline, with knots being selected by visual inspection at the inflection points of the curve. We then estimated the age and sex prevalence of RLS based on the above main model. After this step, we generated the global numbers of people affected by RLS in 2019 (the ‘global RLS envelope’) by multiplying the age- and sex-specific prevalence of RLS by corresponding population data in 2019 from the United Nations Population Division [20]. Then, when estimating the regional prevalence of RLS in H-SDI and LM-SDI countries, we added in the ‘main model’ the term of SDI region, as well as interaction terms of *age* × SD*I* region and *female proportion* × SD*I region* to facilitate the varying relation between age, sex and RLS across H-SDI and LM-SDI regions.

#### Meta-analysis of associated factors of RLS

Because of the intrinsic heterogeneity between epidemiological studies, we chose a random-effects (DerSimonian and Laird method) meta-analysis a priori to explore the effects of major associated factors of RLS [21]. As a rule, we analysed only factors that shared similar definitions and had been investigated in at least three individual articles on the basis of a multivariable analysis. We checked for between-study heterogeneity by Cochran’s Q test and *I*^2^ statistic. The *I*^2^ represents the percentage of total variation across studies because of true between-study differences rather than chance. An *I*^2^ of >75% indicates substantial heterogeneity [22].

#### Estimation of the regional prevalence and cases of RLS in 2019

Then, we classified the world into ten SDI-WHO regions to account for development and geography simultaneously. An ‘associated factor-based model,’ as adopted in our series of papers on global health metrics, was used to distribute the global RLS cases into ten SDI-WHO regions [23–25] using the formula:



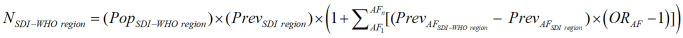



where *N_SDI_* _−_ *_WHO region_* refers to the cases of RLS in each of the ten SDI-WHO regions, *Pop_SDI_* _−_ *_WHO region_* is the age- and sex-specific *de facto* population of people in 2019 in each SDI-WHO region, *Prev_SDI_*  *_region_* indicates the estimated age- and sex-specific prevalence of RLS in 2019 in H-SDI or LM-SDI where the corresponding SDI-WHO region belongs. *AF_1_* − *AF_n_* are the selected associated factors. *Prev_AF SDI_* _−_ *_WHO region_* is the prevalence of selected associated factors in each SDI-WHO region and *Prev_AF SDI region_* is the prevalence of selected associated factors in H-SDI or LM-SDI where this SDI-WHO region stands. *OR_AF_* is the estimated/synthesised OR of a specific associated factor.

We included diabetes, smoking, and depression in the model as associated factors. We obtained the prevalence estimates of diabetes in 2014 and current smoking in 2018 from the WHO Global Health Observatory data repository [26], and the prevalence of depression among people aged 20–79 years in 2019 from the GBD 2019 Study [19]. We generated the prevalence of RLS in the ten SDI-WHO regions by dividing the number of RLS cases by the mid-year population aged 20 to 79 years.

We conducted all analyses in R, version 3.3.0 (R Core Team, Vienna, Austria) and Stata, version 14.0 (StataCorp LLC, College Station, Texas, USA). The significance level was set as *P* < 0.05 in two-sided tests for all analyses.

## RESULTS

**Figure 1:**
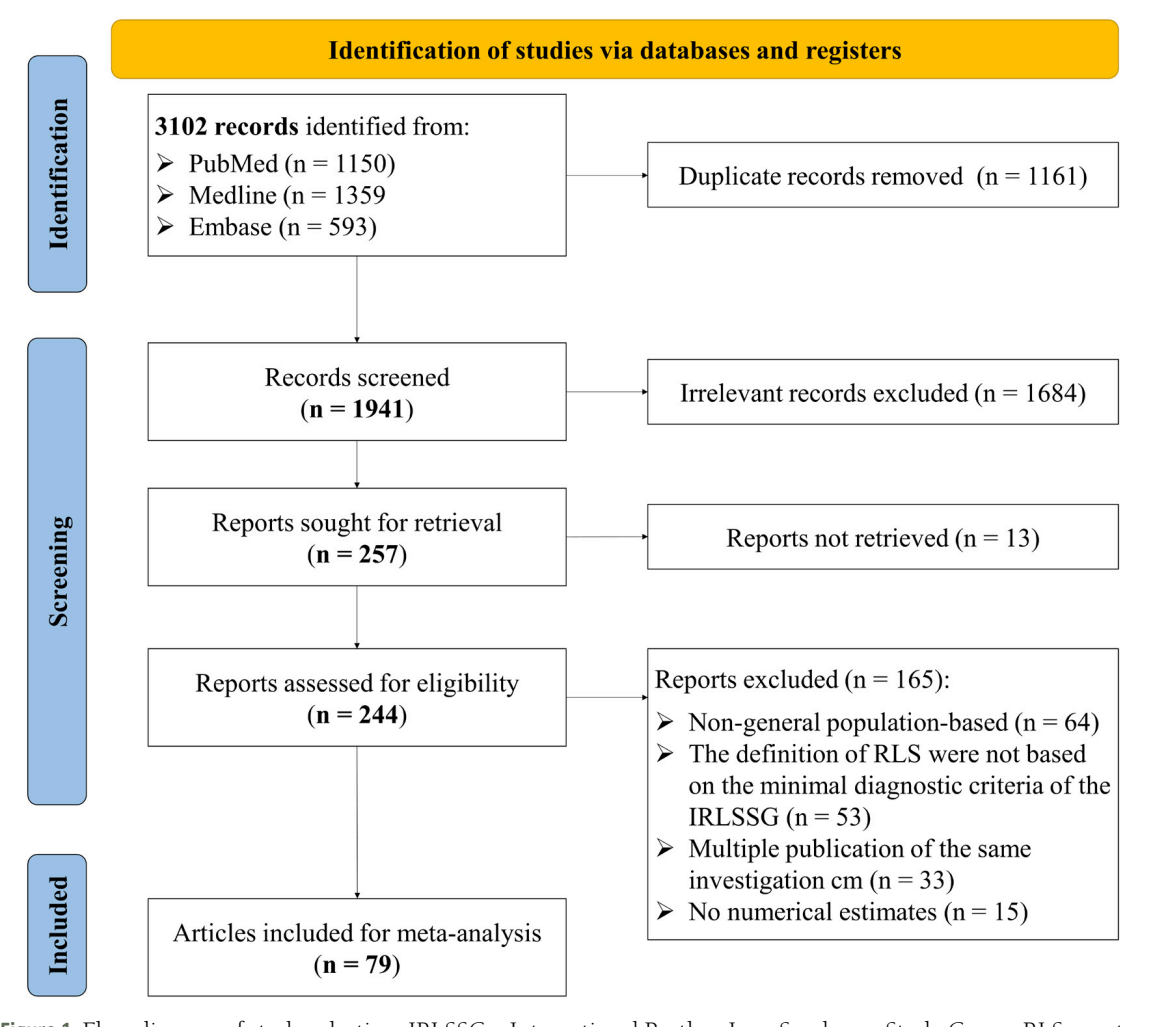
Flow diagram of study selection. IRLSSG – International Restless Legs Syndrome Study Group, RLS – restless leg syndrome.

**Figure 2:**
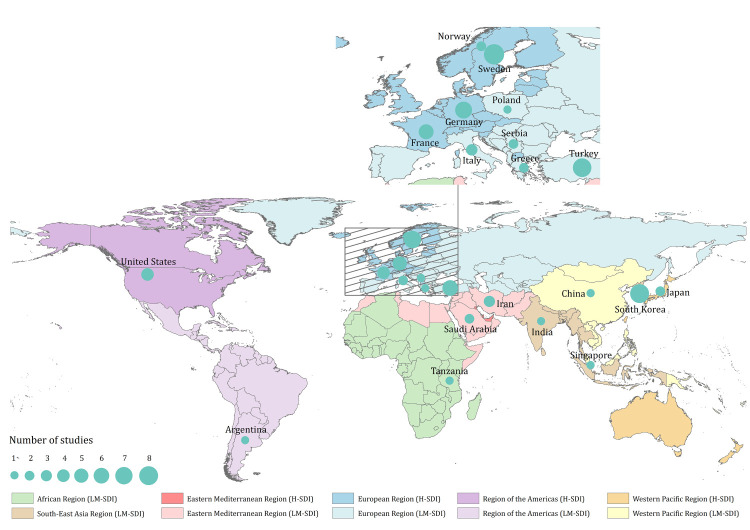
Location of included articles reporting the prevalence of RLS. H-SDI – high socio-demographic index countries, LM-SDI – low- and middle socio-demographic index countries.

We retrieved 3102 records from the bibliographic databases and screened the full text of 257 for eligibility (**Figure 1**). Ultimately, 52 articles met the inclusion criteria and provided prevalence estimates for RLS in the general population using the minimal diagnostic criteria set by IRLSSG without frequency, duration and/or severity restrictions (Table S5 in the **Online Supplementary Document**). The selected studies were published between 2000 and 2023, covering 15768 RLS cases from 228301 participants across 23 countries (**Figure 2**; Table S5 in the **Online Supplementary Document**). In terms of study quality, 49 (94%) of the 52 included articles were assessed to have a quality score of 6 or above (Table S6 in the **Online Supplementary Document**).

Based on the extracted data points (126 in total), we explored the sex-specific relations between age and RLS prevalence in H-SDI and LM-SDI regions (Figure S1 in the **Online Supplementary Document**). In H-SDI countries, there was a consistent increase in RLS prevalence with advancing age, although this trend plateaued after the age of 60 years. The pattern was different in LM-SDI countries, where the prevalence of RLS increased from early adulthood, peaked at approximately 60 years, and then demonstrated a slight decrease thereafter. Notably, the prevalence of RLS in females was consistently higher than that in males, across H-SDI and LM-SDI regions.

**Figure 3:**
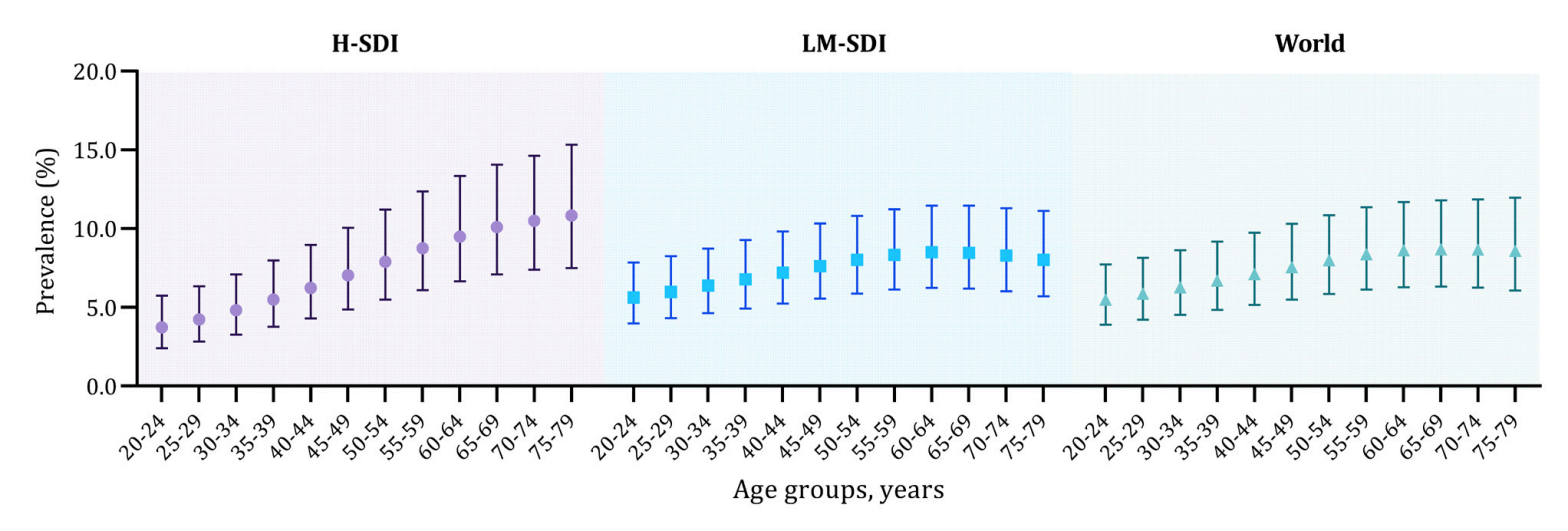
The age-specific prevalence of RLS globally and across SDI regions in 2019.

After adjusting for the demographic profile in 2019, the global prevalence of RLS in people aged 20–79 years was 7.12% (95% CI = 5.15–9.76), with a higher prevalence in females compared to males (8.27%; 95% CI = 6.00–11.28 vs 5.98%; 95% CI = 4.30–8.25) (**Figure 3**, **Table 1**). In 2019, a total of 356.07 million (95% CI = 257.61–488.09) people aged 20–79 years were affected by RLS, with females accounting for 57.89% of these cases. The prevalence was comparable between H-SDI and LM-SDI regions (7.29%; 95% CI = 5.04–10.41 vs 7.10%; 95% CI = 5.16–9.70), yet the absolute number of affected individuals was greater in LM-SDI countries (nearly tenfold) given their much larger population size (323.06 million, 95% CI = 234.79–440.97 vs 33.02 million; 95% CI = 22.82–47.12) (Table S7–8 in the **Online Supplementary Document**).

**Table 1:** Estimated prevalence and number of cases of RLS in people aged 20–79 years worldwide in 2019, by age group and sex

	Prevalence of RLS, % (95% CI)			People with RLS in millions (95% CI)		
**Age group in years**	**Male**	**Female**	**Overall**	**Male**	**Female**	**Overall**
20–24	4.67 (3.30–6.58)	6.39 (4.54–8.94)	5.50 (3.90–7.72)	14.25 (10.06–20.09)	18.28 (13.00–25.57)	32.53 (23.06–45.65)
25–29	4.98 (3.56–6.93)	6.82 (4.91–9.41)	5.87 (4.22–8.13)	15.08 (10.78–20.98)	19.47 (14.02–26.87)	34.55 (24.81–47.85)
30–34	5.31 (3.82–7.35)	7.27 (5.27–9.96)	6.27 (4.53–8.63)	16.26 (11.70–22.48)	21.33 (15.46–29.21)	37.59 (27.16–51.70)
35–39	5.66 (4.08–7.80)	7.74 (5.63–10.58)	6.69 (4.84–9.17)	15.48 (11.15–21.35)	20.57 (14.94–28.09)	36.04 (26.09–49.44)
40–44	6.02 (4.34–8.29)	8.24 (5.99–11.23)	7.12 (5.16–9.74)	14.85 (10.71–20.45)	19.93 (14.49–27.15)	34.78 (25.21–47.60)
45–49	6.39 (4.62–8.76)	8.75 (6.39–11.87)	7.56 (5.50–10.30)	15.25 (11.03–20.91)	20.61 (15.05–27.96)	35.86 (26.08–48.87)
50–54	6.75 (4.90–9.22)	9.24 (6.78–12.48)	8.00 (5.84–10.85)	14.87 (10.80–20.30)	20.42 (14.97–27.56)	35.28 (25.77–47.86)
55–59	7.05 (5.12–9.62)	9.67 (7.09–13.04)	8.37 (6.11–11.35)	13.41 (9.74–18.30)	18.72 (13.73–25.24)	32.13 (23.46–43.55)
60–64	7.23 (5.24–9.89)	9.94 (7.27–13.42)	8.62 (6.28–11.70)	11.25 (8.15–15.38)	16.22 (11.87–21.90)	27.47 (20.02–37.29)
65–69	7.25 (5.24–9.93)	9.99 (7.29–13.50)	8.68 (6.31–11.79)	9.25 (6.69–12.67)	13.90 (10.16–18.80)	23.15 (16.84–31.47)
70–74	7.17 (5.14–9.91)	9.96 (7.22–13.55)	8.67 (6.26–11.87)	6.19 (4.44–8.55)	10.00 (7.25–13.61)	16.19 (11.68–22.16)
75–79	7.03 (4.93–9.90)	9.81 (6.97–13.61)	8.58 (6.07–11.97)	3.81 (2.67–5.36)	6.70 (4.76–9.29)	10.51 (7.43–14.66)
Overall (20–79)	5.98 (4.30–8.25)	8.27 (6.00–11.28)	7.12 (5.15–9.76)	149.93 (107.93–206.84)	206.14 (149.68–281.26)	356.07 (257.61–488.09)

CI – confidence interval, RLS – restless leg syndrome

**Figure 4:**
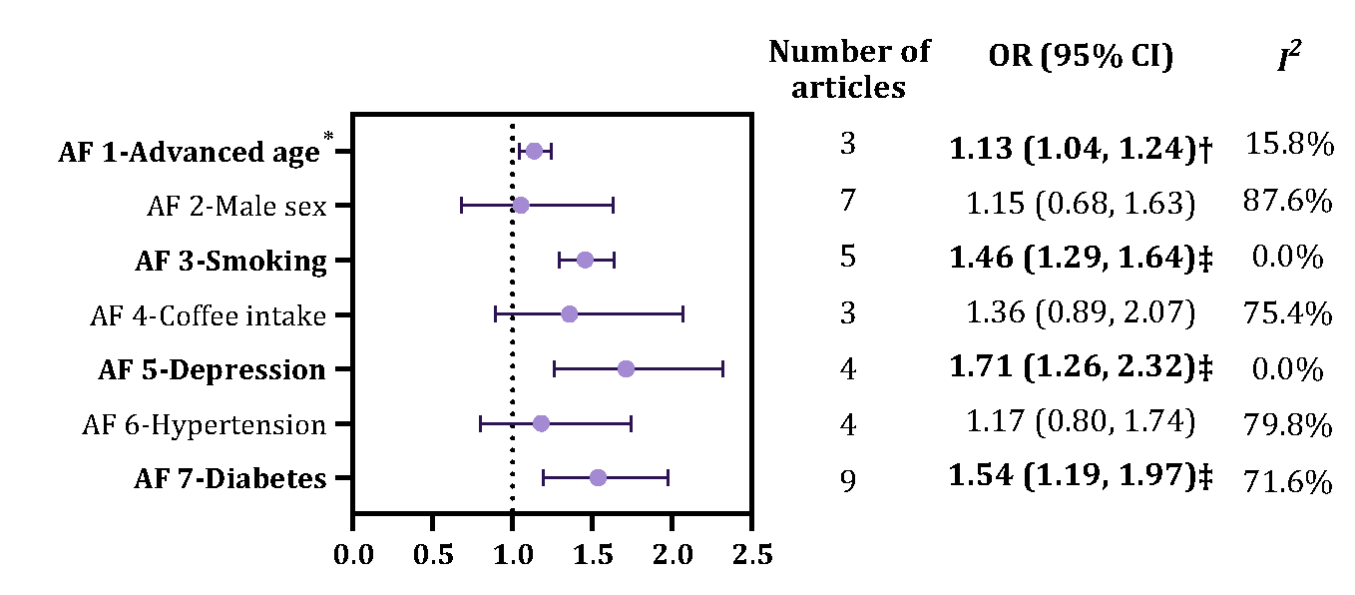
Synthesised effect size of 7 associated factors of RLS. All models were based on random-effects meta-analysis; those factors were investigated in at least three articles using multivariable analysis. AF – associated factors, CI – confidence interval, OR – odds ratio. *A per-decade increase. †*P* < 0.05. ‡*P* < 0.001.

Due to limited data availability and heterogenous definitions across studies, we evaluated seven potential factors associated with RLS in the meta-analysis. Advanced age, smoking, depression, and diabetes were revealed to be significantly associated with RLS, with pooled ORs of 1.13 (95% CI = 1.04–1.24), 1.46 (95% CI = 1.29–1.64), 1.71 (95% CI = 1.26–2.32), and 1.54 (95% CI = 1.19–1.97), respectively, through random-effects meta-analyses (**Figure 4**). Contributing articles for every factor and the detailed corresponding process of those meta-analyses are provided in Table S9 in the **Online Supplementary Document**.

**Figure 5:**
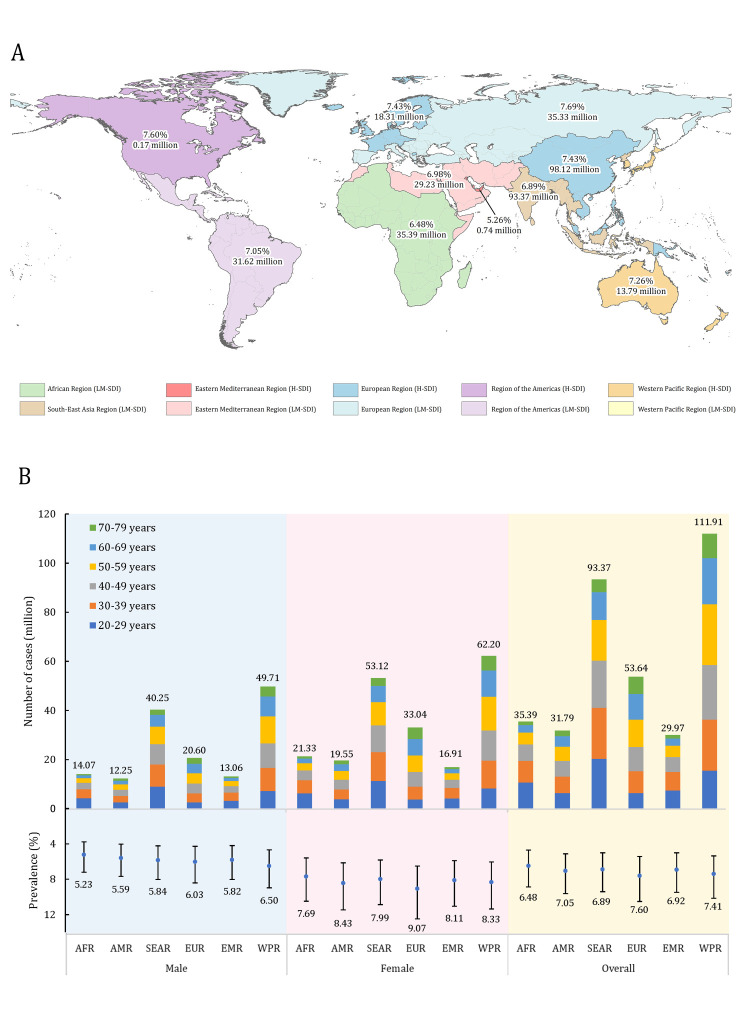
The regional prevalence and number of people with RLS in 2019. AFR – African Region, AMR – Region of the Americas, EMR – Eastern Mediterranean Region, EUR – European Region, H-SDI – high socio-demographic index countries, LM-SDI – low and middle socio-demographic index countries, SEAR – Southeast Asia Region, WPR – Western Pacific Region.

Among WHO regions in 2019, the prevalence of RLS was the highest in EUR (7.60% [95% CI = 5.44-10.52]), but the lowest in AFR (6.48%; 95% CI = 4.70–8.87) (**Figure 5**; Table S10 in the **Online Supplementary Document**). Remarkably, WPR had the largest share of global RLS cases (111.91 million; 95% CI = 80.93–153.42 (31.43% of 356.07 million)), while EMR had the smallest proportion (29.97 million; 95% CI = 21.72–41.05 (8.42%)). The 20–29-year-old age group contributed the most cases of RLS for AFR; the 30–39-year-old age group for AMR, SEAR, and EMR; and the 50–59-year-old age group for EUR and WPR.

## DISCUSSION

In this comprehensive systematic review and modelling study, we incorporated data from 52 population-based studies spanning 23 countries to estimate the global burden of RLS among people aged 20–79 years in 2019. Employing the minimal diagnostic criteria established by the IRLSSG, we estimated a prevalence of 7.12% (95% CI = 5.15–9.76), translating to approximately 356.07 million (95% CI = 257.61–488.09) individuals affected by RLS worldwide. We observed RLS was consistently more prevalent among females than males throughout adulthood. Intriguingly, while H-SDI and LM-SDI countries showed comparable prevalence rates, most cases were concentrated in LM-SDI countries, largely due to the population size disparities. This warrants further investigation into health care access and management in different sociodemographic contexts. Factors such as advanced age, smoking, depression, and diabetes were identified as positively associated with RLS. Geographically, the prevalence was the highest in EUR (7.60%; 95% CI = 5.44–10.52) and the lowest in AFR (6.48%; 95% CI = 4.70–8.87). Notably, WPR accounted for the largest share of global RLS cases, whereas EMR had the smallest share in 2019.

This study, to the best of our knowledge, is the first to report the global and regional prevalence and number cases of RLS using IRLSSG's widely accepted case definitions [9,10]. Our estimated prevalence is more than double that reported in the prior meta-analysis by Broström et al. (7.12% vs 3%) [14]. Such a considerable discrepancy likely arises from the historical heterogeneity in RLS diagnostic criteria, such as the absence of standardisation in phrasing essential diagnostic criteria [1,10]. This lack of standardisation has led to variations in symptom frequency, duration, and severity criteria across different studies, which may have further contributed to the observed discrepancy in RLS prevalence. During the selection process, we limited the inclusion of studies to those that ascertained RLS using the IRLSSG's minimal diagnostic criteria, but without frequency, duration and/or severity restrictions. Given that the pooled estimate by Broström et al. [14] was derived from a heterogeneous mixture of studies based on various definition, among which some put additional limitations on frequency, duration, and severity, it is therefore not surprising to observe a relatively higher than expected RLS prevalence in our estimation.

The substantial variability in reported RLS prevalence across age groups underscores the imperative for age-specific prevalence estimations [4,27], which is a methodological refinement that stands as a principal strength of our study. Through extracting multiple data points from each included article, we constructed a hierarchical data set that provides a more detailed perspective than prior studies, many of which relied on single estimates from individual studies [14]. As demonstrated in our meta-regression analysis, RLS was more common in older people than in younger people, thereby reinforcing the perception of RLS as a progressive chronic condition [28]. However, this age-prevalence relationship varies across SDI regions. In H-SDI countries, the prevalence of RLS consistently increases throughout adulthood, while in LM-SDI countries, it peaks at around 60 years and then slowly decreases. Such a different pattern across development regions might be attributed to greater mortality risks among the elderly in less developed regions. In all instances, RLS prevalence was consistently higher in females than in males, which is in accordance with existing literature and underscores the need for gender-specific research to understand the pathophysiology and clinical management of RLS [4,13,29,30]. This sex disparity might be resulted by a combination of factors, such as increased exposure to risk factors, including oestrogen and iron deficiency, and a higher prevalence of comorbid conditions like fibromyalgia, among females [31–33].

Another key feature of our study is that the effects of seven individual factors associated with RLS were investigated by meta-analysis. Our rigorous selection criteria included only those studies reporting ORs from multivariable analyses, thereby mitigating the potential biases often associated with univariable analyses. The assessment of these associated factors of RLS has noteworthy clinical implications. Notably, advanced age was found to be positively associated with RLS, a finding consistent with our meta-regression analysis and reflective of the broader pattern observed in neurodegenerative disorders, where prevalence commonly escalates with increasing age [1,10]. Smoking emerged as another behavioural factor associated with RLS, potentially reflecting underlying neurobiological pathways or circulatory complications exacerbated by tobacco use, which warrant further mechanistic studies and the promotion of smoking cessation interventions [16,34]. Moreover, our analysis identified depression and diabetes as comorbidities significantly associated with RLS. The positive association between RLS and depression necessitates a proactive clinical stance on sleep disorder evaluations in patients with mood disorders, given the profound influence of sleep on mental well-being [6,35]. Similarly, the link with diabetes emphasises the intricate interplay between metabolic health and neurological conditions, suggesting that RLS screening might be prudent in diabetic populations [36].

In this study, the highest prevalence of RLS among individuals aged 20–79 years was observed in EUR, with the lowest prevalence noted in AFR, a variation likely influenced by the demographic structures in those regions. In addition, WPR accounted for the greatest number of RLS cases, while EMR reported the fewest, a trend attributable to the substantial differences in population size between these regions. Taken together, the regional disparity in both prevalence and absolute number of cases, as previously described in our methods section, was largely a combined result of demography and the uneven distribution of associated factors. The age-specific estimates of RLS cases indicate a comparatively younger demographic structure in AFR. With the global ageing process continuing in the next several decades, a considerable increasing trend in both RLS prevalence and case numbers is likely to be seen. Moreover, other major associated factors of RLS, especially smoking, diabetes, and depression, are expected to rise significantly worldwide. The large and increasing burden of RLS highlights that RLS should not remain a neglected health issue and more efforts to improve prevention, early diagnosis, and treatment of RLS should be strengthened, as should awareness of the disease among health care providers and the general public [1,10].

Several limitations of our study should also be acknowledged. First, although we used the unified definitions of RLS, substantial heterogeneity among the included studies existed. The sources of heterogeneity could be partially dissected by multivariable meta-analysis, yet only a limited set of cluster-level variables were examined, leaving room for unidentified confounding factors. Second, the estimation of global and regional prevalence and cases of RLS was inherently shaped by the demographic structure (age and sex) and an associated factor-based model that only included three associated factors (current smoking, diabetes, and depression) of RLS, and other explanatory variables were not accounted for. Notably, genetic factors, which have been suggested to play a role in RLS and exhibit variability across ethnicities, were not explored due to the lack of relevant data from the studies we included [37,38]. Third, our analysis did not extend to estimating the prevalence of RLS in children or provide a national-level breakdown, owing to the paucity of data for these subgroups. Finally, by relying on published, peer-reviewed studies, our study may have overlooked information from unpublished or grey literature, potentially introducing some degree of publication bias into our estimation.

Despite inherent variations, our estimation is of significant policy and research implications. It is crucial to recognise that RLS cases identified in epidemiological studies might not always retain this diagnosis following more thorough examinations in clinical settings. Healthy people might meet the IRLSSG minimal diagnostic criteria yet not exhibit true RLS, especially in the absence of neurological examinations [39]. Key RLS symptoms such as restlessness, akathisia, or sensory misperception in the extremities also occur in many neurological disorders, making it challenging to distinguish them from genuine restless legs symptoms [1,40]. Moreover, several conditions may closely ‘mimic’ RLS symptoms, requiring attention to differential diagnosis. The symptoms, clinical significance, and any mimicking conditions of RLS should be accurately evaluated through an expert face-to-face interview [41]. However, large population-based studies predominantly rely on validated questionnaires. Additionally, not all studies provided a breakdown of RLS prevalence by frequency, duration, or severity. When provided, the reporting and presentation were largely inconsistent [14]. Without detailed evaluation of RLS and consideration of clinical significance, a substantial number of RLS cases across epidemiological studies might be ‘overdiagnoses.’ Perception of RLS/WED as a frivolous, ‘lifestyle’ condition undermines the profound distress experienced by those affected, thereby emphasising the importance of defining ‘clinically significant’ RLS/WED. It is important to clarify that our estimation does not indicate a need for clinical intervention. Nevertheless, the prevalence and case numbers we present are still meaningful as even sporadic RLS symptoms are associated with increased mental health challenges, including anxiety, depression and, in some cases, suicide attempts. Such figures serve to inform stakeholders of the magnitude of this public health problem, highlighting the need for increased awareness and resource allocation to address this often-overlooked condition.

## CONCLUSIONS

We found a substantial global burden of RLS, with the WPR being most notably impacted by this disease. The large number of people affected by RLS symptoms underscores the potential for a considerable public health challenge, especially in the context of an aging global population. The associations of smoking, diabetes, and depression with RLS highlight the need for integrated public health strategies that address these risk factors. Moreover, our findings emphasise the importance of enhancing epidemiological research, particularly in under-studied regions, to close data gaps and refine public health responses. Future research should prioritise expanding geographic coverage and conducting longitudinal studies to explore causative factors of RLS.

## Additional material


Online Supplementary Document
