# Supplementary material for: The global and regional prevalence of restless legs syndrome among adults: A systematic review and modelling analysis
Source: J Glob Health. 2024 Jun 7;14:04113. doi: 10.7189/jogh.14.04113 (PMC11156251; doi:10.7189/jogh.14.04113)
Supplement: Online Supplementary Document [file jogh-14-04113-s001.pdf]

Supplementary Appendix

Song et al. The global and regional prevalence of restless legs syndrome among adults: a systematic review and modelling analysis

Table S1. Search strategies to identify studies reporting the prevalence of RLS in the general population

Table S2. The time lag between investigation and publication in the included articles

Table S3. Quality assessment scale for rating the risk of bias

Table S4. Age- and sex-adjusted meta-regression models of cluster-level factors related to the prevalence of RLS (logit form)

Table S5. Detailed characteristics of the included articles (n=52)

Table S6. Quality scores for assessing the risk of bias in the included articles (n=52)

Table S7. Estimated age- and sex-specific prevalence of RLS in 2019, by SDI region (%)

Table S8. Estimated age- and sex-specific cases of RLS in 2019, by SDI region (million)

Table S9. Meta-analyses of associated factors of RLS

Table S10. Estimated regional prevalence and number of cases of RLS in people aged 20–79 years in 2019, by sex

Figure S1. The relations of age and prevalence of RLS, by SDI region

eReferences

This supplementary material has been provided by the authors to give readers additional information about their work.

Table S1. Search strategies to identify studies reporting the prevalence of RLS in the general population

Database	Access date	Search terms
**PubMed**	12th Nov, 2023	(Restless legs syndrome[Title/Abstract] OR Restless leg syndrome [Title/Abstract] OR Willis-Ekbom disease[Title/Abstract] OR RLS[Title/Abstract] OR Ekbom syndrome [Title/Abstract]) AND (prevalence [Title/Abstract] OR epidemiology [Title/Abstract])<br>Filter: Humans
**MEDLINE (1950-)**	12th Nov, 2023	1 exp Restless Legs Syndrome/<br>2 Restless legs syndrome.ab,ti.<br>3 Restless leg syndrome.ab,ti.<br>4 Willis-Ekbom disease.ab,ti.<br>5 RLS.ab,ti.<br>6 Ekbom syndrome.ab,ti.<br>7 (prevalen* or epidemiolog*).ab,ti.<br>8 1 or 2 or 3 or 4 or 5 or 6<br>9 7 and 8<br>10 limit 9 to (humans)
**EMBASE (1980-)**	12th Nov, 2023	1 'restless legs syndrome'/exp<br>2 'restless legs syndrome':ab,ti<br>3 'restless leg syndrome':ab,ti<br>4 'Willis-Ekbom disease':ab,ti<br>5 'RLS':ab,ti<br>6 'Ekbom syndrome':ab,ti<br>7 'prevalence'/exp<br>8 'epidemiology'/exp<br>9 prevalence:ab,ti<br>10 epidemiology:ab,ti<br>11 #1 OR #2 OR #3 OR #4 OR #5 OR #6<br>12 #7 OR #8 OR #9 OR #10<br>13 #11 AND #12<br>14 #13 AND [embase]/lim NOT ([embase]/lim AND [medline]/lim) AND 'human'/de AND ('article'/it OR 'article in press'/it OR 'note'/it)

Table S2. The time lag between investigation and publication in the included articles

Study ID	Author(s)	Year of publication	Year of investigation	Time-lag (year)
R1[1]	Szentkirályi, A. et al.	2019	2010	9
R1[1]	Szentkirályi, A. et al.	2019	2014.5	4.5
R2[2]	Bagheri, R., et al.	2018	2015	3
R3[3]	Fereshtehnejad, S.M., et al.	2017	2011.5	5.5
R4[4]	Stehlik, R., et al.	2017	NA	NA
R5[5]	Cho, C.H., et al.	2017	NA	NA
R6[6]	Sherbin, N., et al.	2017	NA	NA
R7[7]	Safak, E.D., et al.	2016	2013	3
R8[8]	Kubo, K., et al.	2016	2013	3
R9[9]	Cho, S.J., et al.	2015	2011.5	3.5
R10[10]	Altunayoglu, C.V., et al.	2015	NA	NA
R11[11]	Güler, S., et al.	2015	2013	2
R12[12]	Rist, P.M., et al.	2015	2010	5
R13[13]	Xue, R., et al.	2015	2014.5	0.5
R14[14]	Wali, S.O., et al.	2015	2013	2
R15[15]	Rist, P.M., et al.	2014	2000	14
R16[16]	Burtscher, C., et al.	2014	2010.5	3.5
R17[17]	Stehlik, R., et al.	2014	NA	NA
R18[18]	Giannini, G., et al.	2014	2011.5	2.5
R19[19]	Szentkiralyi, A., et al.	2014	2013	1
R20[20]	Pekmezovic, T., et al.	2013	2011.5	1.5
R21[21]	Kim, W.H., et al.	2013	2008	5
R22[22]	Sawanyawisuth, K., et al.	2013	2008	5
R23[23]	Yilmaz, N.H., et al.	2013	2006	7
R24[24]	Yun, C.H., et al	2012	2005.5	6.5
R25[25]	Kim, W.H., et al.	2012	2008	4
R26[26]	Allen, R.P., et al.	2011	2007	4
R27[27]	Wesström, J., et al.	2010	2006.5	3.5
R28[28]	Park, Y.M., et al.	2010	NA	NA
R29[29]	Kim, K.W., et al.	2010	2005.5	4.5
R30[30]	Taşdemir, M., et al.	2010	2006	4
R31[31]	Cho, S.J., et al.	2009	2006.5	2.5
R32[32]	Persi, G.G., et al.	2009	2006.5	2.5
R33[33]	Broman, J.E.,et al.	2008	NA	NA
R34[34]	Happe, S., et al	2008	2003	5
R35[35]	Hadjigeorgiou, G.M., et al.	2007	NA	NA
R36[36]	Rangarajan, S.,et al.	2007	2005	2
R37[37]	Ulfberg, J., et al.	2007	NA	NA
R38[38]	McCrink, L., et al.	2007	NA	NA
R39[39]	Lee, H.B., et al.	2006	2004	2
R40[40]	Mizuno, S., et al.	2005	2000.5	4.5
R41[41]	Allen, R.P., et al.	2005	NA	NA
R42[42]	Högl, B., et al.	2005	2000	5
R43[43]	Bjorvatn, B., et al.	2005	NA	NA
R44[44]	Berger, K., et al.	2004	2000	4
R45[45]	Ulfberg, J., et al.	2001	NA	NA
R46[46]	Ulfberg, J., et al.	2001	1999	2
R47[47]	Tan, E.K., et al.	2001	NA	NA
R48[48]	Rothdach, A.J., et al.	2000	1997.5	2.5
R49[49]	Pienczk-Ręcławowicz K, et al.	2022	NA	NA
R50[50]	Aksoy S, et al.	2021	2019	2
R51[51]	Johnson DA, et al.	2020	2014	6
R52[52]	Sadeghniiat-Haghighi, K., et al.	2023	2018.5	4.5

Note: RLS= Restless Legs Syndrome; NA=Not available; The average time-lag between investigation and publication was 3.98 based on 64 articles with available data.

Table S3. Quality assessment scale for rating the risk of bias

Bias type	Low risk (score=2)	Moderate risk (score=1)	High risk (score=0)
Selection (sample population)	1) Sample from the general population, not a select group;<br>2) Consecutive unselected population;<br>3) Rationale for case and control selection explained.	1) Sample selected from large population but selection criteria not defined;<br>2) Sample selection ambiguous but may be representative;<br>3) Rationale for cases and controls not explained;<br>4) Eligibility criteria not explained;<br>5) Analysis to adjust for sampling strategy bias.	1) Highly select population making it difficult to generalise finding;<br>2) Sample selection ambiguous and sample unlikely to be representative.
Selection (sample size)	1) Sample size calculation performed and adequate.	1) Sample size calculation performed and reasons for not meeting sample size given;<br>2) Sample size calculation not performed but all eligible persons studied.	1) Sample size estimation unclear or only sub-sample studied.
Selection (participation rate)	1) High response rate (>85%).	1) Moderate response rate (70-85%).	1) Low response rate (<70%);<br>2) Response rate not reported.
Performance bias (outcome assessment)	1) Diagnosis using consistent criteria and direct examination.	1) Assessment from administrative database or register;<br>2) Assessment from hospital record or interviewer.	1) Assessment from non-validated data or generic estimate from the overall population.
Performance bias (analytical methods to control for bias)	1) Analysis appropriate for the type of sample (subgroup analysis/regression etc.).	1) Analysis does not account for common adjustment.	1) Data confusing.

Table S4. Age- and sex-adjusted meta-regression models of cluster-level factors related to the prevalence of RLS (logit form)

Moderator	Number of articles	Number of data points	β	95 % CI		P value
Age	49	126	0.0158	0.0125	0.0192	<0.0001
Female proportion	49	126	0.331	0.2616	0.4003	<0.0001
Sex						
Female	25	43	Reference			
Male	20	42	-0.3387	-0.4082	-0.2693	<0.0001
Setting						
Mixed	36	87	Reference			
Urban	13	49	-0.3281	-0.6842	0.0279	0.0708
SDI	49	126	0.8279	-3.2588	4.9146	0.6913
SDI Region						
H-SDI	24	66	Reference			
LM-SDI	19	60	-0.0068	-0.6358	0.6223	0.9832
WHO region						
EUR	23	75	Reference			
EMR	4	9	0.2323	-0.3761	0.8407	0.4543
WPR	10	32	-1.0417	-1.5245	-0.5589	<0.0001
AMR	3	4	0.4748	-0.2441	1.1936	0.1955
Investigation year	49	126	-0.0073	-0.0388	0.0242	0.6492

Note: RLS= Restless Legs Syndrome; CI=confidence interval; SDI= Socio-demographic Index; H-SDI=High-SDI countries; LM-SDI= low- and middle-SDI countries; EUR=European Region; EMR=Eastern Mediterranean Region; WPR= Western Pacific Region; AMR=Region of the Americas.

Table S5. Detailed characteristics of the included articles (n=52)

ID	Author	Year Published	Country	WHO region	SDI	Setting	Investigation Date	Study design	Diagnostic criteria	Based on?	No. sample	Age range (years)	Female proportion	Cases
R1[1]	Szentkirályi, A. et al.	2019	Germany	EUR	0.878; 0.888	Mixed; Urban	2008-2012; 2013-2016	Cohort	The minimal criteria published by the IRLSSG	Interview	1107; 247	20-81; 35-70	0.46; 0.49	186; 14
R2[2]	Bagheri, R., et al.	2018	Iran	EMR	0.649	Urban	2014-2016	Cross-sectional	International RLS Scale	Questionnaire, interview	980	NS	1.00	157
R3[3]	Fereshtehnejad, S.M., et al.	2017	Iran	EMR	0.63	Urban	2011-2012	Cross-sectional	IRLSSG consensus diagnostic features	Face-to-face Interview	19176	30+	0.66	1580
R4[4]	Stehlik, R., et al.	2017	Sweden	EUR	0.858	Mixed	NA	Cohort	4 minimal diagnostic criteria of the IRLSSG	Questionnaire	2727	18-64	1.00	194
R5[5]	Cho, C.H., et al.	2017	Republic of Korea	WPR	0.855	Mixed	NA	Cohort	4 minimal diagnostic criteria of the IRLSSG	Questionnaire	3026	40-69	0.46	142
R6[6]	Sherbin, N., et al.	2017	Saudi Arabia	EMR	0.76	Urban	NA	Cross-sectional	IRLSSG	Questionnaire	2095	18+	0.40	309
R7[7]	Safak, E.D., et al.	2016	Turkey	EUR	0.707	Urban	2013	Cross-sectional	4 essential criteria of the IRLSSG	Face-to-face interview	665	60+	0.47	105
R8[8]	Kubo, K., et al.	2016	Japan	WPR	0.855	Mixed	2013	Cross-sectional	4 minimal diagnostic criteria of the IRLSSG	Face-to-face Interview	985	NS	0.62	10
R9[9]	Cho, S.J., et al.	2015	Republic of Korea	WPR	0.846	Mixed	2011-2012	Cross-sectional	4 questions based on the IRLSSG criteria published in 2003	Door-to-door visit and face-to-face interview	2695	19-69	0.50	142
R10[10]	Altunayoglu, C.V., et al.	2015	Turkey	EUR	0.689	Mixed	NA	Cross-sectional	4 minimal criteria of the IRLSSG	Questionnaire, interview and physical and neurological examination	3789	20+	0.57	169
R11[11]	Güler, S., et al.	2015	Turkey	EUR	0.707	Mixed	2013	Cross-sectional	4 diagnostic criteria defined by IRLSSG (2003)	Questionnaire	4003	18+	0.49	282
R12[12]	Rist, P.M., et al.	2015	France	EUR	0.806	Urban	2008-2012	Cohort	4 minimal diagnostic criteria of the IRLSSG	Interview	2070	65+	0.65	417
R13[13]	Xue, R., et al.	2015	China	WPR	0.654	Mixed	2014-2015	Cross-sectional	4 diagnostic criteria defined by IRLSSG (2003)	Questionnaire and interview	6437	8-17	0.46	141
R14[14]	Wali, S.O., et al.	2015	Saudi Arabia	EMR	0.76	Mixed	2013	Cross-sectional	4 questions suggested by the IRLSSG	Questionnaire and interview	2682	30-60	0.52	224

ID	Author	Year Published	Country	WHO region	SDI	Setting	Investigation Date	Study design	Diagnostic criteria	Based on?	No. sample	Age range (years)	Female proportion	Cases
R15[15]	Rist, P.M., et al.	2014	France	EUR	0.777	Urban	1999-2001	Cohort	4 minimal diagnostic criteria of the IRLSSG	Interview	1035	65+	0.63	218
R16[16]	Burtscher, C., et al.	2014	Tanzania	AFR	0.354	Mixed	2010-2011	Cross-sectional	The essential diagnostic criteria of the IRLSSG	Questionnaire	28606	14+	NS	156
R17[17]	Stehlik, R., et al.	2014	Sweden	EUR	0.849	Mixed	NA	Cross-sectional	4 minimal diagnostic criteria of the IRLSSG	Questionnaire	3060	18-64	1.00	774
R18[18]	Giannini, G., et al.	2014	Italy	EUR	0.782	Mixed	2011-2012	Cross-sectional	4 minimal diagnostic criteria of the IRLSSG	Face-to-face Interview	1709	18+	0.57	170
R19[19]	Szentkiralyi, A., et al.	2014	Germany	EUR	0.886	Mixed	2013	Cohort	The minimal criteria of the IRLSSG	Face-to-face Interview with a short questionnaire	1311	25-75	0.53	96
R20[20]	Pekmezovic, T., et al.	2013	Serbia	EUR	0.735	Both	2011-2012	Cross-sectional	4 minimal diagnostic criteria of the IRLSSG (2003)	Face-to-face interview	2112	18+	0.52	107
R21[21]	Kim, W.H., et al.	2013	Republic of Korea	WPR	0.833	Both	2008	Cross-sectional	4-item RLS Questionnaire	Face-to-face Interview	1985	65+	0.59	199
R22[22]	Sawanyawisuth, K., et al.	2013	USA	AMR	0.819	Mixed	2007-2009	Cross-sectional	4 minimal diagnostic criteria of the IRLSSG	Telephone interview and questionnaire	1754	18+	0.56	253
R23[23]	Yilmaz, N.H., et al.	2013	Turkey	EUR	0.641	Mixed	2005-2007	Cross-sectional	4 minimal diagnostic criteria of the IRLSSG	Questionnaire and face-to-face interview	706	18-98	0.61	39
R24[24]	Yun, C.H., et al	2012	Republic of Korea	WPR	0.817	Mixed	2005-2006	Cohort	IRLSSG	Face-to-face interviews	3365	NS	0.48	150
R25[25]	Kim, W.H., et al.	2012	Republic of Korea	WPR	0.833	Both	2008	Cross-sectional	The Korean version of IRLSSG and 5 essential diagnostic criteria	Face-to-face interviews	1990	65+	NS	202
R26[26]	Allen, R.P., et al.	2011	USA	AMR	0.814	Mixed	2007	Cross-sectional	4 RLS diagnostic criteria by email	Questionnaires	61792	NS	NS	4484
R27[27]	Wesström, J., et al.	2010	Sweden	EUR	0.84	Mixed	2006-2007	Cross-sectional	Standardized criteria of the IRLSSG	Questionnaires and telephone interviews	5000	25-64	1.00	551
R28[28]	Park, Y.M., et al.	2010	Republic of Korea	WPR	0.823	Mixed	NA	Cohort	Modified IRLSSG criteria	Questionnaires	1000	40-69	1.00	65
R29[29]	Kim, K.W., et al.	2010	Republic of Korea	WPR	0.817	Urban	2005-2006	Cohort	4 minimal diagnostic criteria for RLS recommended by NIH	Face-to-face interviews by two psychiatrists	714	65+	0.58	59
R30[30]	Taşdemir, M., et al.	2010	Turkey	EUR	0.641	Rural	2006	Cross-sectional	The Turkish version of 4 minimal criteria	Questionnaires and face-to-face, door-to-door	2111	18+	0.52	72

ID	Author	Year Published	Country	WHO region	SDI	Setting	Investigation Date	Study design	Diagnostic criteria	Based on?	No. sample	Age range (years)	Female proportion	Cases
									suggested by the IRLSSG	interviews and neurological examinations				
R31[31]	Cho, S.J., et al.	2009	Republic of Korea	WPR	0.823	Mixed	2006-2007	Cross-sectional	Korean translation of the 4 features of RLS defined by the IRLSSG	Face-to-face interviews	6509	18-64	0.60	72
R32[32]	Persi, G.G., et al.	2009	Argentina	AMR	0.653	Both	2006-2007	Cross-sectional	3 questions of the 4 essential IRLSSG criteria and a modified questionnaire	Questionnaires	471	18-91	0.60	95
R33[33]	Broman, J.E.,et al.	2008	Sweden	EUR	0.835	Mixed	NA	Cross-sectional	4-question set by the IRLSSG	Questionnaires	1335	20-59	0.56	251
R34[34]	Happe, S., et al	2008	Germany	EUR	0.858	Urban	2003	Cross-sectional	4 minimal diagnostic criteria of the IRLSSG	Face-to-face interviews	1312	25-75	0.53	96
R35[35]	Hadjigeorgiou, G.M., et al.	2007	Greece	EUR	0.753	Urban	NA	Cross-sectional	The Greek version of the 4 minimal IRLSSG clinical criteria	Interviews with trained medical doctors	3033	20+	0.53	117
R36[36]	Rangarajan, S.,et al.	2007	India	SEAR	0.429	Mixed	2005	Cross-sectional	4 essential National Institutes of NIH/IRLSSG criteria	Questionnaires by face-to-face interviews	1266	18-90	0.45	27
R37[37]	Ulfberg, J., et al.	2007	Sweden	EUR	0.832	Mixed	NA	Cross-sectional	The minimal criteria provided by the IRLSSG	Telephone interviews	1000	18-90	0.51	50
R38[38]	McCrink, L., et al.	2007	USA	AMR	0.809	Mixed	NA	Cross-sectional	4 diagnostic questions for RLS	Face-to-face interviews (Europe) or telephone interviews (USA)	6014	18+	0.61	454
R39[39]	Lee, H.B., et al.	2006	USA	AMR	0.811	Mixed	2004	Cohort	7-item RLS Questionnaire	Questionnaires	1028	Mean: 58.11	NS	42
R40[40]	Mizuno, S., et al.	2005	Japan	WPR	0.826	Urban	2000-2001	Cross-sectional	4 essential symptoms defined by the IRLSSG	Mailed questionnaires and face-to-face interviews	3287	65+	0.54	150
R41[41]	Allen, R.P., et al.	2005	Europe and USA	EUR	France-0.781; Germany-0.853; Italy-0.757; Spain-0.709;	Mixed	NA	Cross-sectional	4 diagnostic questions for RLS based on standard diagnostic criteria	Face-to-face interviews	15391	18+	NS	1114

ID	Author	Year Published	Country	WHO region	SDI	Setting	Investigation Date	Study design	Diagnostic criteria	Based on?	No. sample	Age range (years)	Female proportion	Cases
					UK-0.793;USA-0.802									
R42[42]	Högl, B., et al.	2005	Italy	EUR	0.753	Mixed	2000	Cross-sectional	4 minimal criteria of the IRLSSG (1995)	Face-to-face interviews and examinations	701	50-89	0.52	74
R43[43]	Bjorvatn, B., et al.	2005	Norway and Denmark	EUR	0.861; 0.848	Mixed	NA	Cross-sectional	The updated diagnostic criteria	Telephone interviews	1927	18+	0.51	222
R44[44]	Berger, K., et al.	2004	Germany	EUR	0.847	Mixed	1997	Cross-sectional	The IRLSSG criteria	Face-to-face interviews	4107	20-79	0.51	433
R45[45]	Ulfberg, J., et al.	2001	Sweden	EUR	0.811	Urban	NA	Cross-sectional	4 minimal diagnostic criteria of the IRLSSG (1995)	Questionnaires and telephone contacts	2608	18-64	0.00	181
R46[46]	Ulfberg, J., et al.	2001	Sweden	EUR	0.819	Urban	1999	Cross-sectional	The 4 symptom questions from the IRLSSG as minimal diagnostic criteria	Questionnaires	140	18-64	1.00	16
R47[47]	Tan, E.K., et al.	2001	Singapore	WPR	0.749	Mixed	NA	Cross-sectional	4 minimal criteria of the IRLSSG	Clinical examinations and interviews	157	55-93	0.56	1
R48[48]	Rothdach, A.J., et al.	2000	Germany	EUR	0.844	Mixed	1997-1998	Cohort	4 minimal standard criteria of the IRLSSG (1995)	Face-to-face interviews	369	65-83	0.47	36
R49[49]	Pienczk-Ręcławowicz K, et al.	2022	Poland	EUR	0.798	Mixed	NA	Cross-sectional	Questionnaire with IRLSSG's Scale	Clinical examinations and interviews	2097	13-18	0.56	21
R50[50]	Aksoy S, et al.	2021	Turkey	EUR	0.748	Urban	2018-2019	Cross-sectional	The Turkish version of the 4 minimum criteria from IRLSSG	Face-to-face interviews	622	18+	0.50	75
R51[51]	Johnson DA, et al.	2020	USA	AMR	0.845	Mixed	2013-2016	Cohort	the International Restless Legs Screening Questionnaire	Clinical examinations and interviews	795	Average: 63.1	0.67	200
R52[52]	Sadeghniiat-Haghighi, K., et al.	2023	Iran	EMR	0.665	Both	2017-2020	Cross-sectional	IRLSSG consensus diagnostic features	Face-to-face interviews	3198	Mean: 39.7	0.50	374

Note: RLS= Restless Legs Syndrome; SDI= Socio-demographic Index; AFR= African Region; EUR=European Region; EMR=Eastern Mediterranean Region; WPR= Western Pacific Region; AMR=Region of the Americas; SEAR= Southeast Asia Region; IRLSSG= International Restless Legs Syndrome Study Group; NIH= National Institute of Health.

Table S6. Quality scores for assessing the risk of bias in the included articles (n=52)

ID	Author	Year Published	Quality score					
			Sample population	Sample size	Participation	Outcome assessment	Analytical methods	Total scores
R1[1]	Szentkirályi, A. et al.	2019	2	1	0	2	1	6
R2[2]	Bagheri, R., et al.	2018	2	2	2	2	2	10
R3[3]	Fereshtehnejad, S.M., et al.	2017	2	1	2	2	2	9
R4[4]	Stehlik, R., et al.	2017	2	1	0	2	1	6
R5[5]	Cho, C.H., et al.	2017	2	1	0	2	2	7
R6[6]	Sherbin, N., et al.	2017	2	1	2	2	2	9
R7[7]	Safak, E.D., et al.	2016	2	1	2	2	2	9
R8[8]	Kubo, K., et al.	2016	1	1	2	2	1	7
R9[9]	Cho, S.J., et al.	2015	2	0	0	2	2	6
R10[10]	Altunayoglu, C.V., et al.	2015	2	2	2	2	2	10
R11[11]	Güler, S., et al.	2015	2	1	2	2	2	9
R12[12]	Rist, P.M., et al.	2015	2	1	2	2	2	9
R13[13]	Xue, R., et al.	2015	2	1	2	2	2	9
R14[14]	Wali, S.O., et al.	2015	2	1	0	2	2	7
R15[15]	Rist, P.M., et al.	2014	2	0	0	2	2	6
R16[16]	Burtscher, C., et al.	2014	2	1	0	2	1	6
R17[17]	Stehlik, R., et al.	2014	2	1	0	2	2	7
R18[18]	Giannini, G., et al.	2014	1	1	0	2	2	6
R19[19]	Szentkiralyi, A., et al.	2014	2	1	0	2	2	7
R20[20]	Pekmezovic, T., et al.	2013	2	1	2	2	2	9
R21[21]	Kim, W.H., et al.	2013	2	1	0	2	1	6
R22[22]	Sawanyawisuth, K., et al.	2013	2	1	0	2	2	7
R23[23]	Yilmaz, N.H., et al.	2013	2	2	2	2	2	10
R24[24]	Yun, C.H., et al	2012	2	1	0	2	2	7
R25[25]	Kim, W.H., et al.	2012	2	1	0	2	2	7
R26[26]	Allen, R.P., et al.	2011	2	1	0	2	1	6
R27[27]	Wesström, J., et al.	2010	2	1	2	2	1	8
R28[28]	Park, Y.M., et al.	2010	2	1	1	2	2	8
R29[29]	Kim, K.W., et al.	2010	1	1	0	2	2	6
R30[30]	Taşdemir, M., et al.	2010	2	2	2	2	2	10
R31[31]	Cho, S.J., et al.	2009	2	1	1	2	2	8
R32[32]	Persi, G.G., et al.	2009	2	1	2	1	1	7
R33[33]	Broman, J.E.,et al.	2008	2	1	0	1	1	5
R34[34]	Happe, S., et al	2008	2	1	0	2	2	7
R35[35]	Hadjigeorgiou, G.M., et al.	2007	2	1	1	2	2	8
R36[36]	Rangarajan, S.,et al.	2007	2	2	2	2	2	10
R37[37]	Ulfberg, J., et al.	2007	1	1	0	2	1	5
R38[38]	McCrink, L., et al.	2007	1	1	0	1	1	4
R39[39]	Lee, H.B., et al.	2006	1	1	1	2	2	7
R40[40]	Mizuno, S., et al.	2005	2	1	0	2	2	7
R41[41]	Allen, R.P., et al.	2005	2	1	0	2	2	7
R42[42]	Högl, B., et al.	2005	2	1	2	2	2	9

ID	Author	Year Published	Quality score					
			Sample population	Sample size	Participation	Outcome assessment	Analytical methods	Total scores
R43[43]	Bjorvatn, B., et al.	2005	2	1	0	2	2	7
R44[44]	Berger, K., et al.	2004	2	1	0	2	2	7
R45[45]	Ulfberg, J., et al.	2001	2	1	0	2	1	6
R46[46]	Ulfberg, J., et al.	2001	2	0	1	2	1	6
R47[47]	Tan, E.K., et al.	2001	2	1	0	2	1	6
R48[48]	Rothdach, A.J., et al.	2000	2	1	0	2	2	7
R49[49]	Pienczk-Ręcławowicz K, et al.	2022	2	1	0	2	2	7
R50[50]	Aksoy S, et al.	2021	2	2	0	2	2	8
R51[51]	Johnson DA, et al.	2020	2	2	0	2	2	8
R52[52]	Sadeghniiat-Haghighi, K., et al.	2023	2	2	0	2	2	8

Table S7. Estimated age- and sex-specific prevalence of RLS in 2019, by SDI region (%)

Age group	H-SDI			LM-SDI		
	Male	Female	Overall	Male	Female	Overall
20-24 years	2.90 (1.86-4.50)	4.64 (3.01-7.09)	3.73 (2.41-5.75)	4.78 (3.39-6.71)	6.50 (4.63-9.05)	5.61 (3.99-7.84)
25-29 years	3.31 (2.18-5.00)	5.28 (3.53-7.85)	4.24 (2.82-6.34)	5.10 (3.66-7.06)	6.92 (5.00-9.51)	5.98 (4.31-8.25)
30-34 years	3.77 (2.53-5.58)	6.00 (4.08-8.74)	4.83 (3.27-7.09)	5.43 (3.92-7.48)	7.36 (5.36-10.05)	6.38 (4.62-8.74)
35-39 years	4.28 (2.91-6.27)	6.80 (4.68-9.79)	5.50 (3.77-7.97)	5.77 (4.18-7.93)	7.82 (5.70-10.64)	6.78 (4.93-9.27)
40-44 years	4.86 (3.32-7.06)	7.69 (5.32-10.97)	6.24 (4.30-8.96)	6.13 (4.44-8.40)	8.29 (6.05-11.25)	7.20 (5.24-9.81)
45-49 years	5.49 (3.76-7.93)	8.65 (6.02-12.26)	7.04 (4.87-10.05)	6.48 (4.71-8.84)	8.76 (6.42-11.83)	7.61 (5.56-10.33)
50-54 years	6.16 (4.24-8.86)	9.67 (6.76-13.62)	7.90 (5.48-11.21)	6.81 (4.98-9.26)	9.20 (6.78-12.36)	8.01 (5.88-10.81)
55-59 years	6.82 (4.70-9.77)	10.67 (7.48-14.95)	8.74 (6.09-12.35)	7.08 (5.17-9.60)	9.55 (7.04-12.81)	8.33 (6.12-11.22)
60-64 years	7.40 (5.12-10.55)	11.54 (8.12-16.07)	9.50 (6.64-13.35)	7.21 (5.25-9.80)	9.72 (7.15-13.06)	8.50 (6.23-11.47)
65-69 years	7.83 (5.45-11.10)	12.18 (8.63-16.84)	10.08 (7.10-14.07)	7.16 (5.21-9.76)	9.66 (7.09-13.00)	8.46 (6.19-11.45)
70-74 years	8.12 (5.64-11.51)	12.61 (8.93-17.42)	10.49 (7.38-14.64)	6.97 (5.04-9.56)	9.41 (6.86-12.75)	8.29 (6.02-11.28)
75-79 years	8.31 (5.67-11.97)	12.90 (8.98-18.07)	10.84 (7.49-15.33)	6.70 (4.74-9.37)	9.05 (6.47-12.50)	8.01 (5.71-11.12)
20-79 years	5.60 (3.83-8.11)	9.00 (6.26-12.74)	7.29 (5.04-10.41)	6.02 (4.35-8.26)	8.19 (5.98-11.13)	7.10 (5.16-9.70)

Note: H-SDI=High-SDI countries; LM-SDI= low- and middle-SDI countries.

Table S8. Estimated age- and sex-specific cases of RLS in 2019, by SDI region (million)

Age group	H-SDI			LM-SDI		
	Male	Female	Overall	Male	Female	Overall
20-24 years	0.51 (0.33-0.79)	0.75 (0.49-1.15)	1.27 (0.82-1.95)	13.74 (9.73-19.29)	17.53 (12.51-24.41)	31.27 (22.24-43.71)
25-29 years	0.66 (0.43-0.99)	0.93 (0.62-1.39)	1.59 (1.06-2.38)	14.42 (10.35-19.99)	18.54 (13.40-25.48)	32.96 (23.75-45.47)
30-34 years	0.79 (0.53-1.17)	1.14 (0.77-1.66)	1.92 (1.30-2.82)	15.48 (11.17-21.32)	20.19 (14.69-27.56)	35.67 (25.86-48.87)
35-39 years	0.91 (0.62-1.33)	1.36 (0.93-1.95)	2.27 (1.55-3.29)	14.56 (10.53-20.01)	19.21 (14.00-26.14)	33.77 (24.54-46.15)
40-44 years	1.02 (0.69-1.48)	1.53 (1.06-2.18)	2.55 (1.75-3.66)	13.83 (10.02-18.97)	18.40 (13.43-24.97)	32.23 (23.45-43.94)
45-49 years	1.20 (0.83-1.74)	1.83 (1.27-2.59)	3.03 (2.10-4.33)	14.04 (10.21-19.17)	18.79 (13.78-25.37)	32.83 (23.99-44.54)
50-54 years	1.35 (0.93-1.94)	2.08 (1.45-2.93)	3.43 (2.38-4.87)	13.52 (9.87-18.36)	18.34 (13.52-24.63)	31.85 (23.39-43.00)
55-59 years	1.45 (1.00-2.08)	2.26 (1.58-3.16)	3.71 (2.58-5.24)	11.96 (8.74-16.23)	16.46 (12.14-22.08)	28.42 (20.88-38.31)
60-64 years	1.41 (0.97-2.01)	2.25 (1.58-3.13)	3.66 (2.56-5.14)	9.84 (7.17-13.38)	13.97 (10.28-18.77)	23.81 (17.46-32.15)
65-69 years	1.32 (0.92-1.87)	2.20 (1.56-3.05)	3.52 (2.48-4.92)	7.93 (5.77-10.81)	11.70 (8.59-15.75)	19.63 (14.36-26.55)
70-74 years	1.24 (0.86-1.76)	2.17 (1.53-2.99)	3.41 (2.40-4.76)	4.95 (3.57-6.79)	7.83 (5.71-10.62)	12.78 (9.29-17.40)
75-79 years	0.92 (0.63-1.32)	1.75 (1.22-2.45)	2.67 (1.85-3.78)	2.89 (2.04-4.04)	4.95 (3.54-6.84)	7.84 (5.58-10.88)
20-79 years	12.77 (8.74-18.48)	20.25 (14.09-28.64)	33.02 (22.82-47.12)	137.16 (99.19-188.35)	185.9 (135.60-252.62)	323.06 (234.79-440.97)

Note: H-SDI=High-SDI countries; LM-SDI= low- and middle-SDI countries.

Table S9. Meta-analyses of associated factors of RLS

Associated factor	ID	Author	Year Published	Country	Meta-analysis
**Age-per year increase**					
	R22[22]	Sawanyawisuth, K., et al.	2013	USA	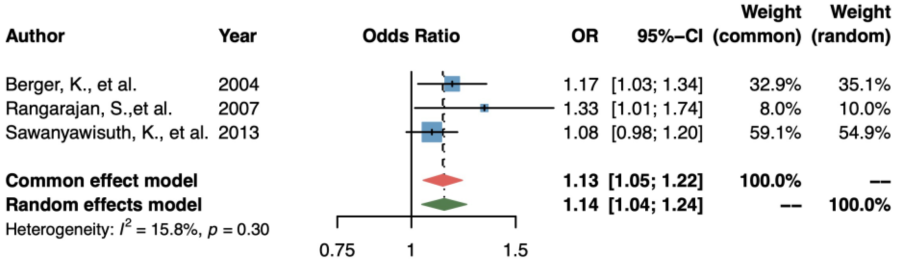
	R36[36]	Rangarajan, S.,et al.	2007	India	
	R44[44]	Berger, K., et al.	2004	Germany	
**Male Sex**					
	R6[6]	Sherbin, N., et al.	2017	Saudi Arabia	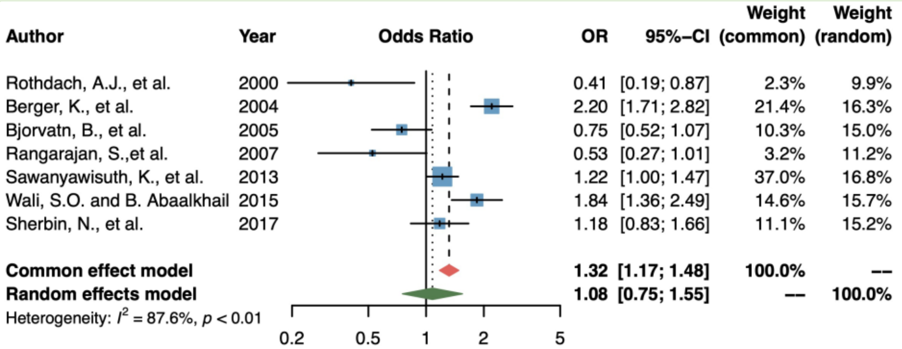
	R14[14]	Wali, S.O., et al.	2015	Saudi Arabia	
	R43[43]	Bjorvatn, B., et al.	2005	Norway and Denmark	
	R48[48]	Rothdach, A.J., et al.	2000	Germany	
	R22[22]	Sawanyawisuth, K., et al.	2013	USA	
	R36[36]	Rangarajan, S.,et al.	2007	India	
	R44[44]	Berger, K., et al.	2004	Germany	
**Smoking**					
	R3[3]	Fereshtehnejad, S.M., et al.	2017	Iran	
	R6[6]	Sherbin, N., et al.	2017	Saudi Arabia	
	R22[22]	Sawanyawisuth, K., et al.	2013	USA	
	R36[36]	Rangarajan, S.,et al.	2007	India	

Associated factor	ID	Author	Year Published	Country	Meta-analysis
	R44[44]	Berger, K., et al.	2004	Germany	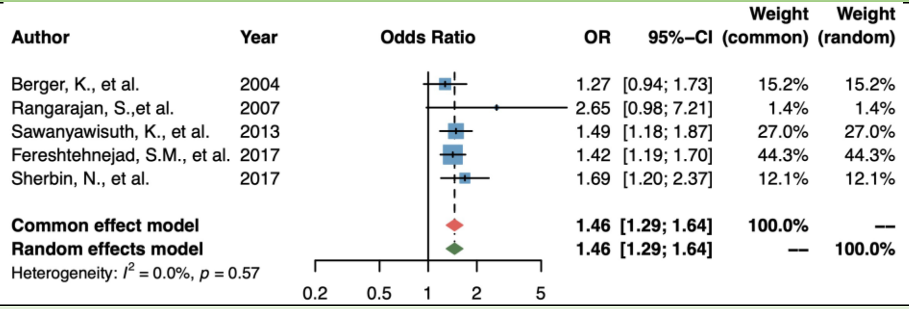
**Coffee intake**					
	R2[2]	Bagheri, R., et al.	2018	Iran	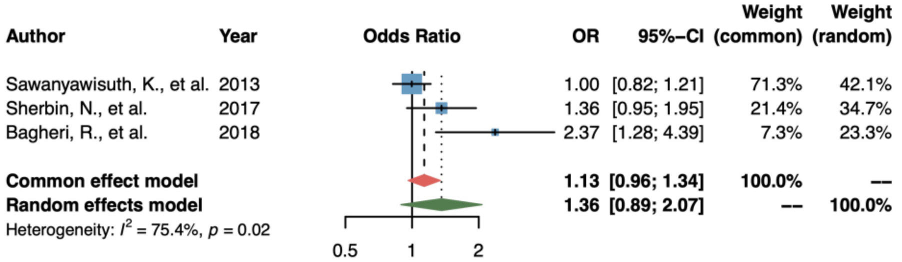
	R6[6]	Sherbin, N., et al.	2017	Saudi Arabia	
	R22[22]	Sawanyawisuth, K., et al.	2013	USA	
**Depression**					
	R6[6]	Sherbin, N., et al.	2017	Saudi Arabia	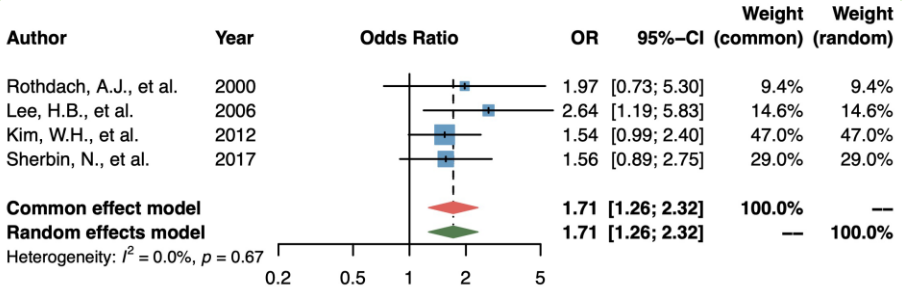
	R25[25]	Kim, W.H., et al.	2012	Republic of Korea	
	R39[39]	Lee, H.B., et al.	2006	USA	
	R48[48]	Rothdach, A.J., et al.	2000	Germany	
**Hypertension**					
	R2[2]	Bagheri, R., et al.	2018	Iran	

Associated factor	ID	Author	Year Published	Country	Meta-analysis
	R6[6]	Sherbin, N., et al.	2017	Saudi Arabia	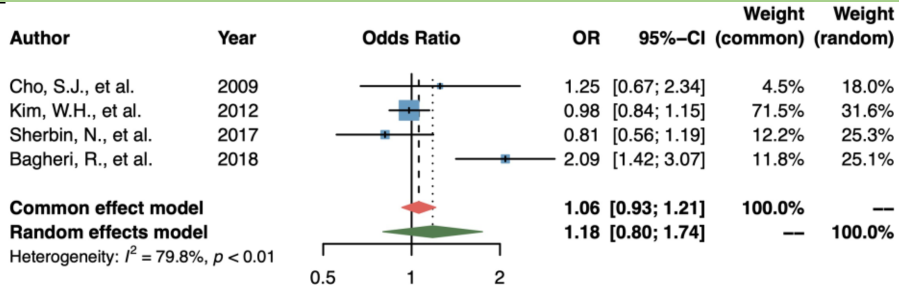
	R25[25,29]	Kim, W.H., et al.	2012	Republic of Korea	
	R31[31]	Cho, S.J., et al.	2009	Republic of Korea	
**Diabetes**					
	R2[2]	Bagheri, R., et al.	2018	Iran	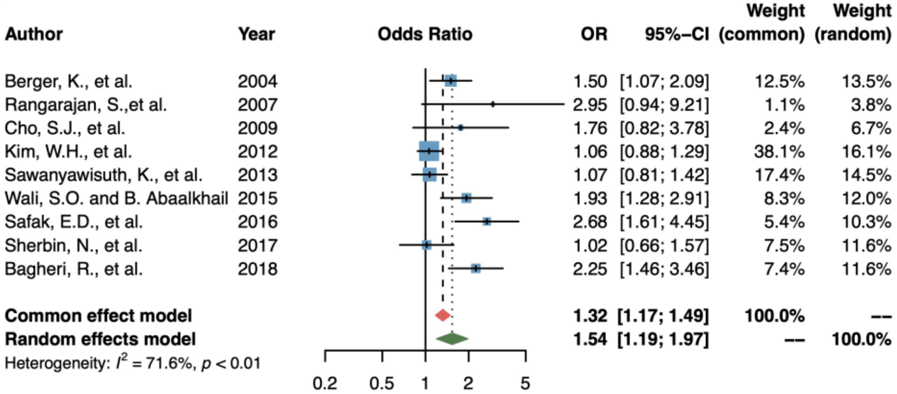
	R6[6]	Sherbin, N., et al.	2017	Saudi Arabia	
	R7[7]	Safak, E.D., et al.	2016	Turkey	
	R14[14]	Wali, S.O. and B. Abaalkhail	2015	Saudi Arabia	
	R25[25]	Kim, W.H., et al.	2012	Republic of Korea	
	R31[31]	Cho, S.J., et al.	2009	Republic of Korea	
	R22[22]	Sawanyawisuth, K., et al.	2013	USA	
	R36[36]	Rangarajan, S.,et al.	2007	India	
	R44[44]	Berger, K., et al.	2004	Germany	

Table S10. Estimated regional prevalence and number of cases of RLS in people aged 20–79 years in 2019, by sex

WHO region	Prevalence of RLS (%)			People with RLS (millions)		
	Male	Female	Overall	Male	Female	Overall
**Africa**	5.23 (3.78-7.21)	7.69 (5.60-10.49)	6.48 (4.70-8.87)	14.07 (10.15-19.37)	21.33 (15.52-29.07)	35.39 (25.66-48.44)
**Americas**	5.59 (4.04-7.68)	8.43 (6.15-11.46)	7.05 (5.12-9.63)	12.25 (8.85-16.83)	19.55 (14.25-26.57)	31.79 (23.10-43.39)
**South-East Asia**	5.84 (4.22-8.03)	7.99 (5.82-10.86)	6.89 (5.01-9.42)	40.25 (29.09-55.30)	53.12 (38.73-72.22)	93.37 (67.82-127.53)
**Europe**	6.03 (4.29-8.42)	9.07 (6.51-12.48)	7.60 (5.44-10.52)	20.60 (14.64-28.77)	33.04 (23.73-45.49)	53.64 (38.38-74.26)
**Eastern Mediterranean**	5.82 (4.19-8.02)	8.11 (5.90-11.05)	6.92 (5.02-9.48)	13.06 (9.41-18.00)	16.91 (12.31-23.05)	29.97 (21.72-41.05)
**Western Pacific**	6.50 (4.68-8.97)	8.33 (6.05-11.36)	7.41 (5.36-10.15)	49.71 (35.78-68.57)	62.20 (45.15-84.85)	111.91 (80.93-153.42)

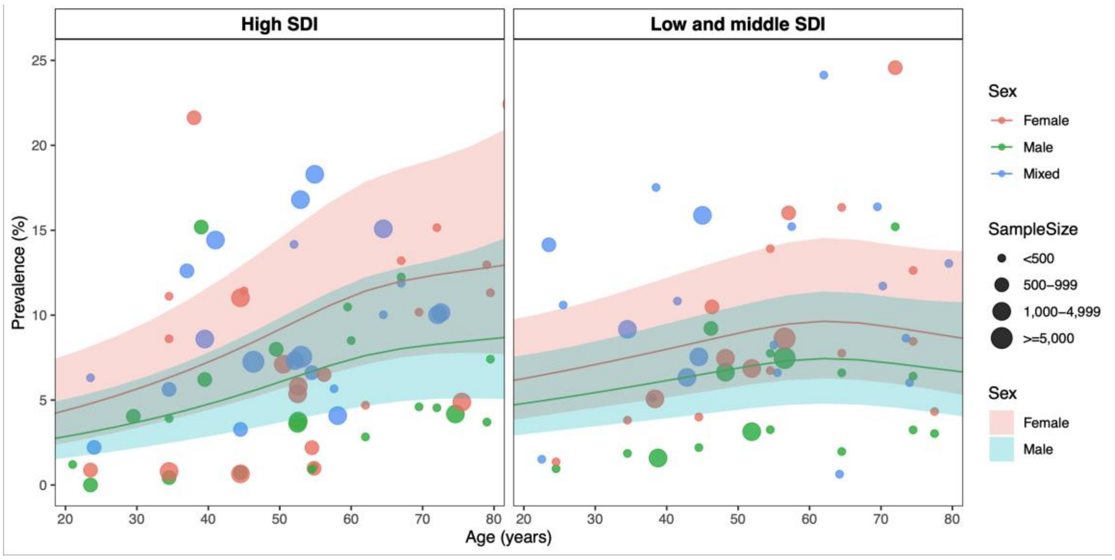

Figure S1. The relations of age and prevalence of RLS, by SDI region

eReference

1. Szentkirályi A, Stefani A, Hackner H, et al. Prevalence and associated risk factors of periodic limb movement in sleep in two German population-based studies. *Sleep* 2019; **42**(3).
2. Bagheri R, Abedi P, Mousavi P, Azimi N. The prevalence of restless legs syndrome and its relationship with demographic characteristics and medical disorders in postmenopausal Iranian women. *Health care for women international* 2018; **39**(12): 1317-25.
3. Fereshtehnejad SM, Rahmani A, Shafieesabet M, et al. Prevalence and associated comorbidities of restless legs syndrome (RLS): Data from a large population-based door-to-door survey on 19176 adults in Tehran, Iran. *PloS one* 2017; **12**(2): e0172593.
4. Stehlik R, Ulfberg J, Zou D, Hedner J, Grote L. Perceived sleep deficit is a strong predictor of RLS in multisite pain - A population based study in middle aged females. *Scandinavian journal of pain* 2017; **17**: 1-7.
5. Cho CH, Kim L, Lee HJ. Individuals with Restless Legs Syndrome Tend to have Severe Depressive Symptoms: Findings from a Community-Based Cohort Study. *Psychiatry investigation* 2017; **14**(6): 887-93.
6. Sherbin N, Ahmed A, Fatani A, et al. The prevalence and associated risk factors of restless legs syndrome among Saudi adults. *Sleep and Biological Rhythms* 2017; **15**(2): 127-35.
7. Safak ED, Gocer S, Mucuk S, et al. The prevalence and related factors of restless leg syndrome in the community dwelling elderly; in Kayseri, Turkey: A cross-sectional study. *Archives of gerontology and geriatrics* 2016; **65**: 29-35.
8. Kubo K, Sugawara N, Kaneda A, et al. Relationship between quality of life and restless legs syndrome among a community-dwelling population in Japan. *Neuropsychiatric disease and treatment* 2016; **12**: 809-15.
9. Cho SJ, Chung YK, Kim JM, Chu MK. Migraine and restless legs syndrome are associated in adults under age fifty but not in adults over fifty: a population-based study. *The journal of headache and pain* 2015; **16**: 554.
10. Altunayoglu Cakmak V, Koc B, Nuhoglu I, et al. Prevalence of restless legs syndrome in Trabzon in the northeast Black Sea Region of Turkey: co-morbidities, socioeconomic factors and biochemical parameters. *Neurological research* 2015; **37**(9): 751-62.
11. Güler S, Caylan A, Nesrin Turan F, Dağdeviren N, Çelik Y. The prevalence of restless legs syndrome in Edirne and its districts concomitant comorbid conditions and secondary complications. *Neurological sciences : official journal of the Italian Neurological Society and of the Italian Society of Clinical Neurophysiology* 2015; **36**(10): 1805-12.
12. Rist PM, Elbaz A, Dufouil C, Tzourio C, Kurth T. Restless Legs Syndrome and Cognitive Function: A Population-based Cross-sectional Study. *The American journal of medicine* 2015; **128**(9): 1023.e33-9.
13. Xue R, Liu G, Ma S, Yang J, Li L. An epidemiologic study of restless legs syndrome among Chinese children and adolescents. *Neurological sciences : official journal of the Italian Neurological Society and of the Italian Society of Clinical Neurophysiology* 2015; **36**(6): 971-6.
14. Wali SO, Abaalkhail B. Prevalence of restless legs syndrome and associated risk factors among middle-aged Saudi population. *Annals of thoracic medicine* 2015; **10**(3): 193-8.
15. Rist PM, Tzourio C, Elbaz A, et al. Structural brain lesions and restless legs syndrome: a cross-sectional population-based study. *BMJ open* 2014; **4**(11): e005938.
16. Burtscher C, Baxmann A, Kassubek J, et al. Prevalence of restless legs syndrome in an urban population of eastern Africa (Tanzania). *Journal of the neurological sciences* 2014; **346**(1-2): 121-7.

17. Stehlik R, Ulfberg J, Hedner J, Grote L. High prevalence of restless legs syndrome among women with multi-site pain: a population-based study in Dalarna, Sweden. *European journal of pain (London, England)* 2014; **18**(10): 1402-9.
18. Giannini G, Zanigni S, Melotti R, et al. Association between restless legs syndrome and hypertension: a preliminary population-based study in South Tyrol, Italy. *European journal of neurology* 2014; **21**(1): 72-8.
19. Szentkirályi A, Völzke H, Hoffmann W, Trenkwalder C, Berger K. Multimorbidity and the risk of restless legs syndrome in 2 prospective cohort studies. *Neurology* 2014; **82**(22): 2026-33.
20. Pekmezovic T, Jovic J, Svetel M, Kostic VS. Prevalence of restless legs syndrome among adult population in a Serbian district: a community-based study. *European journal of epidemiology* 2013; **28**(11): 927-30.
21. Kim WH, Kim BS, Kim SK, et al. Prevalence of insomnia and associated factors in a community sample of elderly individuals in South Korea. *International psychogeriatrics* 2013; **25**(10): 1729-37.
22. Sawanyawisuth K, Palinkas LA, Ancoli-Israel S, Dimsdale JE, Loredo JS. Ethnic differences in the prevalence and predictors of restless legs syndrome between Hispanics of Mexican descent and non-Hispanic Whites in San Diego county: a population-based study. *Journal of clinical sleep medicine : JCSM : official publication of the American Academy of Sleep Medicine* 2013; **9**(1): 47-53.
23. Yilmaz NH, Akbostanci MC, Oto A, Aykac O. Prevalence of restless legs syndrome in Ankara, Turkey: an analysis of diagnostic criteria and awareness. *Acta neurologica Belgica* 2013; **113**(3): 247-51.
24. Yun CH, Lee SK, Kim H, et al. Association between irritable bowel syndrome and restless legs syndrome in the general population. *Journal of sleep research* 2012; **21**(5): 569-76.
25. Kim WH, Kim BS, Kim SK, et al. Restless legs syndrome in older people: a community-based study on its prevalence and association with major depressive disorder in older Korean adults. *International journal of geriatric psychiatry* 2012; **27**(6): 565-72.
26. Allen RP, Bharmal M, Calloway M. Prevalence and disease burden of primary restless legs syndrome: results of a general population survey in the United States. *Movement disorders : official journal of the Movement Disorder Society* 2011; **26**(1): 114-20.
27. Wesström J, Nilsson S, Sundström-Poromaa I, Ulfberg J. Health-related quality of life and restless legs syndrome among women in Sweden. *Psychiatry and clinical neurosciences* 2010; **64**(5): 574-9.
28. Park YM, Lee HJ, Kang SG, et al. Prevalence of idiopathic and secondary restless legs syndrome in Korean Women. *General hospital psychiatry* 2010; **32**(2): 164-8.
29. Kim KW, Yoon IY, Chung S, et al. Prevalence, comorbidities and risk factors of restless legs syndrome in the Korean elderly population - results from the Korean Longitudinal Study on Health and Aging. *Journal of sleep research* 2010; **19**(1 Pt 1): 87-92.
30. Taşdemir M, Erdoğan H, Börü UT, Dilaver E, Kumaş A. Epidemiology of restless legs syndrome in Turkish adults on the western Black Sea coast of Turkey: A door-to-door study in a rural area. *Sleep medicine* 2010; **11**(1): 82-6.
31. Cho SJ, Hong JP, Hahm BJ, et al. Restless legs syndrome in a community sample of Korean adults: prevalence, impact on quality of life, and association with DSM-IV psychiatric disorders. *Sleep* 2009; **32**(8): 1069-76.
32. Persi GG, Etcheverry JL, Vecchi C, Parisi VL, Ayarza AC, Gatto EM. Prevalence of restless legs syndrome: a community-based study from Argentina. *Parkinsonism & related disorders* 2009; **15**(6): 461-5.

33. Broman JE, Mallon L, Hetta J. Restless legs syndrome and its relationship with insomnia symptoms and daytime distress: epidemiological survey in Sweden. *Psychiatry and clinical neurosciences* 2008; **62**(4): 472-5.
34. Happe S, Vennemann M, Evers S, Berger K. Treatment wish of individuals with known and unknown restless legs syndrome in the community. *Journal of neurology* 2008; **255**(9): 1365-71.
35. Hadjigeorgiou GM, Stefanidis I, Dardiotis E, et al. Low RLS prevalence and awareness in central Greece: an epidemiological survey. *European journal of neurology* 2007; **14**(11): 1275-80.
36. Rangarajan S, Rangarajan S, D'Souza GA. Restless legs syndrome in an Indian urban population. *Sleep medicine* 2007; **9**(1): 88-93.
37. Ulfberg J, Bjorvatn B, Leissner L, et al. Comorbidity in restless legs syndrome among a sample of Swedish adults. *Sleep medicine* 2007; **8**(7-8): 768-72.
38. McCrink L, Allen RP, Wolowacz S, Sherrill B, Connolly M, Kirsch J. Predictors of health-related quality of life in sufferers with restless legs syndrome: a multi-national study. *Sleep medicine* 2007; **8**(1): 73-83.
39. Lee HB, Hening WA, Allen RP, Earley CJ, Eaton WW, Lyketsos CG. Race and restless legs syndrome symptoms in an adult community sample in east Baltimore. *Sleep medicine* 2006; **7**(8): 642-5.
40. Mizuno S, Miyaoka T, Inagaki T, Horiguchi J. Prevalence of restless legs syndrome in non-institutionalized Japanese elderly. *Psychiatry and clinical neurosciences* 2005; **59**(4): 461-5.
41. Allen RP, Walters AS, Montplaisir J, et al. Restless legs syndrome prevalence and impact: REST general population study. *Archives of internal medicine* 2005; **165**(11): 1286-92.
42. Högl B, Kiechl S, Willeit J, et al. Restless legs syndrome: a community-based study of prevalence, severity, and risk factors. *Neurology* 2005; **64**(11): 1920-4.
43. Bjorvatn B, Leissner L, Ulfberg J, et al. Prevalence, severity and risk factors of restless legs syndrome in the general adult population in two Scandinavian countries. *Sleep medicine* 2005; **6**(4): 307-12.
44. Berger K, Luedemann J, Trenkwalder C, John U, Kessler C. Sex and the risk of restless legs syndrome in the general population. *Archives of internal medicine* 2004; **164**(2): 196-202.
45. Ulfberg J, Nyström B, Carter N, Edling C. Prevalence of restless legs syndrome among men aged 18 to 64 years: an association with somatic disease and neuropsychiatric symptoms. *Movement disorders : official journal of the Movement Disorder Society* 2001; **16**(6): 1159-63.
46. Ulfberg J, Nyström B, Carter N, Edling C. Restless Legs Syndrome among working-aged women. *European neurology* 2001; **46**(1): 17-9.
47. Tan EK, Seah A, See SJ, Lim E, Wong MC, Koh KK. Restless legs syndrome in an Asian population: A study in Singapore. *Movement disorders : official journal of the Movement Disorder Society* 2001; **16**(3): 577-9.
48. Rothdach AJ, Trenkwalder C, Haberstock J, Keil U, Berger K. Prevalence and risk factors of RLS in an elderly population: the MEMO study. Memory and Morbidity in Augsburg Elderly. *Neurology* 2000; **54**(5): 1064-8.
49. Pienczk-Ręcławowicz K, Pilarska E, Olszewska A, Ręcławowicz D, Konieczna S, Sławek J. The prevalence of the restless legs Syndrome/Willis-Ekbom disease among teenagers, its clinical characteristics and impact on everyday functioning. *Sleep medicine* 2022; **89**: 48-54.
50. Aksoy S, Eker E, Yalcin S, Karaman HIO. Prevalence of restless legs syndrome: a cross-sectional population-based study from northwest of Turkey. *Sleep and Biological Rhythms* 2021; **19**(4): 453-8.

51. Johnson DA, Javaheri S, Guo N, et al. Objective Measures of Sleep Apnea and Actigraphy-Based Sleep Characteristics as Correlates of Subjective Sleep Quality in an Epidemiologic Study: The Jackson Heart Sleep Study. *Psychosomatic medicine* 2020; **82**(3): 324-30.
52. Sadeghniiat-Haghighi K, Akbarpour S, Behkar A, et al. A nationwide study on the prevalence and contributing factors of obstructive sleep apnea in Iran. *Scientific reports* 2023; **13**(1): 17649.
